# Efficacy and Safety of Adding 6 Weeks of Doxycycline to the Essential Package of Care to Treat Filarial Lymphedema: A Double-Blind, Randomized, Controlled Trial in Southern India

**DOI:** 10.4269/ajtmh.24-0337

**Published:** 2024-10-01

**Authors:** Suma Krishnasastry, Anuja Ashok, Ammu Devidas, Sarah Sullivan, Mariana Stephens, Jayla Norman, Elianna Paljug, Andrew Deathe, Andrew Majewski, John Horton, Joseph P. Shott, Ute Klarmann-Schultz, Achim Hoerauf, Eric Ottesen, Charles D. Mackenzie

**Affiliations:** ^1^ WHO Collaborating Centre for Lymphatic Filariasis Morbidity Management and Disability Prevention, Government TD Medical College, Alappuzha, India;; ^2^ Neglected Tropical Disease-Supporting Centre (NTD-SC), Task Force for Global Health, Decatur, Georgia;; ^3^ Tropical Projects, Hitchin, United Kingdom;; ^4^ Division of Neglected Tropical Diseases, Office of Infectious Diseases, Global Health Bureau, US Agency for International Development, Washington, District of Columbia;; ^5^ Institute for Medical Microbiology, Immunology and Parasitology, German Centre for Infection Research (DZIF), University Hospital Bonn, Bonn, Germany;; ^6^ Reaching the Last Mile Fund (RLMF), The Ending Neglected tropical Diseases (END) Fund, New York, New York

## Abstract

Finding additional ways to manage lymphedema due to lymphatic filariasis (LF) is a primary concern for the Global Program to Eliminate Lymphatic Filariasis. The WHO-recommended Essential Package of Care (EPC) consists of skin hygiene, elevation of affected limbs, exercise, protective shoe ware, wound care, and supportive therapy for acute phases. The care program has been successful but often hard to maintain. A double-blind study reexamined previous findings that doxycycline treatment could improve the lymphedematous changes in LF patients. The present study was carried out in a semi-urban location of Kerala, southern India, where *Brugia* sp. is the predominant parasite, and LF mass drug administration had ceased in many areas. Two hundred individuals (aged 14–65 years; 142 females and 58 males) with lymphedema of stages 1–3 were instructed in the EPC and were randomly administered either 200 mg doxycycline or an identical-appearing placebo daily for 6 weeks. Data were collected at 0, 3, 6, 12, 18, and 24 months and included the state of the lymphedema (size, cleanliness, skin thickness and changes), occurrence of adenolymphangitis (ADL) attacks, and patients’ quality of life (QOL). The results demonstrated no difference over time between the two arms of the study; virtually all patients of both groups showed either improvement or “no worsening” in the parameters during the 2-year study period. Importantly, this rigorous trial confirmed that the EPC is of substantial benefit to lymphedema patients by reducing acute ADL and improving their QOL and clinical condition.

## INTRODUCTION

Patients suffering the pathologic consequences of infection with lymphatic filarial parasites may present with major disfiguring changes that include lymphedema with severe dermal changes, hydrocele, and other significant pathologies. More than 40 million people in areas endemic for lymphatic filariasis (LF) suffer from the resulting disabilities and mental stress that these disfigurements usually cause.^1^ Providing appropriate care for these patients is one of the two principal goals of the global effort to eliminate this infection,^2^ and it is a specific requirement for endemic countries seeking to achieve successful WHO validation for having eliminated LF as a public health problem.^3^

The current mainstay for treatment of lymphedema patients living in filariasis-endemic areas is the Essential Package of Care (EPC) recommended by the WHO.^3^^,^^4^ This package includes hygiene practices, skin and wound care (antibiotic/antiseptic creams), exercise, elevation of the limbs, wearing of comfortable and appropriate protective footwear, as well as intermittent antibiotic and antipyretic treatment of adenolymphangitis (ADL) attacks. This relatively simple care package has been successful in improving lymphedema or at least slowing its significant worsening, reducing the frequency of ADL attacks, and improving patients’ quality of life (QoL).^2^^,^^5^ However, a challenge that remains for national LF programs as they continue attempts to provide care for this chronic condition is assisting patients in maintaining the EPC self-care practices over time; many patients stop their self-care activities relatively soon after beginning them for a range of reasons. Consequently, finding more easily applied therapeutic additions to the EPC for lymphedema treatment has long been a goal for those concerned with the care of these patients.

Two earlier studies in Ghana indicated that the tetracycline-class antibiotic, doxycycline, might be an additional pharmacological intervention that could assist in reducing the severity of lymphedema in patients living in LF-endemic areas.^6^^,^^7^ Doxycycline has been used for a wide range of infections, and aside from its well-known effect on bacteria and protozoa, it has also been effective in filarial infections, where its principal lethal action on the worm is to destroy the essential *Wolbachia *sp. endosymbiont that most adult filariae carry.^8^ In these earlier Ghanaian studies, the use of doxycycline for 6 weeks to treat lymphedema stages 2 and 3 resulted in significant reductions in clinical severity after 12 and 24 months. These observations led to suggestions that doxycycline treatment for a period of 6 weeks could improve the clinical status of mild to moderate lymphedema and that patients might therefore benefit from a 6-week course of doxycycline given intermittently (e.g., every other year).^7^

Because the findings of these previous studies could have major implications for the WHO’s recommendations for the Morbidity Management and Disability Prevention (MMDP) arm of the Global Program to Eliminate Lymphatic Filariasis (GPELF), clinical trials were established in five countries^9^ to determine if it would indeed be advantageous to add doxycycline to the EPC. This report describes the results from a study in Kerala, India, with patients living in a semi-urban LF-endemic area where both *Brugia malayi* and *Wuchereria bancrofti* are present,^10^ the former being the dominant filarial species in this study zone.^11^ Evaluation of other patients with filarial lymphedema also took place following identical protocols in Sri Lanka, Mali, Ghana, and Tanzania, whereas an additional site in Cameroon used the same protocol to treat a related lymphedematous condition caused by podoconiosis. The design of these new studies was based on that used in the original Ghanaian studies, but with the addition of new tools and parameters to assess the effects of treatment on both patients’ lymphedema and their QoL.^9^

## MATERIALS AND METHODS

### Study aim.

This double‐blind, randomized, placebo‐controlled study was designed to investigate the impact of 6 weeks of treatment with doxycycline on early (stages 1–3) filarial lymphedema when added to the standard limb hygiene protocol (EPC) recommended by the WHO. The primary objective was to evaluate the effect on the patient’s presenting stage of lymphedema. Secondary objectives included evaluating the effects of this added drug regimen on 1) the circumference and volume of affected limbs, 2) the skin thickness of affected limbs, and 3) the frequency and intensity of acute attacks of ADL. In addition, changes in QoL and an assessment of the tolerability and safety of doxycycline given for 6 weeks were also assessed.

### Study setting.

**Figure 1.**
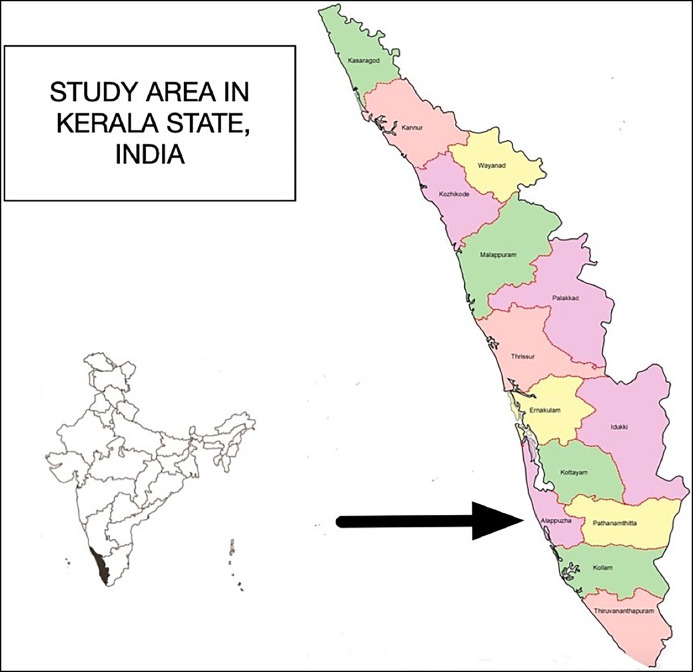
Study settings, Alappuzha, Kerala, India.

The trial was conducted under the guidance of the WHO Filariasis Research Center (FRC) in the Government TD Medical College Hospital, Alappuzha, Kerala, India (Figure 1). Lymphedema patients living either in Kerala’s typical semi-urban areas or along its extensive waterways (canals) were registered for the study. Most of the study subjects were from the Ambalappuzha and Cherthala Taluks regions of Alappuzha, where *B. malayi* is the dominant form of LF.

### Screening procedures.

The potential participants for the study were selected from attendees at the Outpatient Department of the FRC or from their homes in the recruitment area: All were prescreened for suitability to enter the full trial.^9^ After their informed consent for pretrial screening, a detailed history was taken that included demographic data, history of lymphedema and ADL attacks, and relevant details on mass drug administration (MDA) and concomitant medications they might have taken. The physical examination included vital signs, staging of lymphedema, and the presence or absence of hydrocele. All subjects had the following laboratory tests: a standard complete blood profile (i.e., red blood cells, white blood cells, platelets, hemoglobin, and hematocrit), renal function tests (serum creatinine/blood urea nitrogen), liver function tests (aspartate aminotransferase/alanine aminotransferase/gamma-glutamyl transferase), and a serum or urine pregnancy test for women of childbearing potential.

As part of the screening process, all patients, regardless of whether they were subsequently included in the actual trial, were then instructed on the use of the EPC for MMDP, which focuses particularly on rigorous hygiene measures including 1) cleaning of the affected and nonaffected limbs daily with soap and water; 2) keeping the affected limb dry; 3) clipping the nails; 4) using appropriate antibiotics for any ADL episodes per advice from the investigating team; 5) applying antifungal ointment to webs between the toes, nails, and sides of the feet and inside the folds every night; 6) avoiding injuries; 7) elevation of the affected limb when possible; 8) limb exercises; and 9) use of appropriate footwear. Each participant was given soap, towels, plastic bowls, a bucket for washing the limbs, and a diary for recording any ADL attacks that might develop. They were also supplied with antifungal and antiseptic ointments whenever needed. All of these materials were replenished when required.

### Enrollment.

After data acquired during the screening procedures were reviewed, patients were selected for enrollment in the drug study based on the inclusion and exclusion criteria.^9^ The exclusion criteria included being a resident in the endemic area for <5 years, inability to appropriately carry out the EPC, pregnancy or breastfeeding in women, evidence of hepatic, renal or neurological diseases, or having a history of adverse drug reaction to doxycycline or another tetracycline. In addition, women of childbearing potential were counseled on avoiding pregnancy during the treatment period in a culturally appropriate manner, as defined by the local institutional review board. Individuals selected for participation were enrolled within 1 month of the original screening procedures after providing their informed consent for participation in the study. The baseline assessment parameters were then repeated (medical history, physical examination, stage of lymphedema, any concomitant medications, etc., as listed above). If the interval between screening and enrollment was more than 1 month, the blood tests originally carried out during the initial screening were repeated. Other procedures carried out on the day of enrollment included 1) measurement of four-point limb circumference^9^^,^^12^; 2) ultrasound-based measurement of the thickness of the skin over the lateral and medial malleolus of the affected limb and of the normal limb, if available; 3) measurement of volume and circumference of each limb using the LymphaTech^®^ three-dimensional photoscan technique^13^; 4) clinical photographs of the affected and normal limbs; and 5) questionnaires administered by trained social scientist/healthcare workers to assess both their history of ADL attacks and their QoL. The QoL was measured through the WHODAS 2.0 12-item version.^14^ The status of limb hygiene was judged and recorded independently by a trained nurse following a defined list of parameters.

### Trial procedures.

The selected participants were men and women 14–65 years old who had leg lymphedema of stages 1–3.^15^ The enrollees were given repeated training sessions on lymphedema management (the EPC) to ensure that they were well-informed about the recommended procedures prior to the first drug administration. The participants were then randomized to receive either doxycycline or placebo following a provided randomization table. Based on their lymphedema stages, the participants were segregated into group A (stages 1–3) or group B (stages 4–6)^15^ (Note: Findings from group B with its smaller number of patients [*N* = 20] are not included in the current report).

Daily doxycycline 200 mg or placebo was given to all patients for a period of 6 weeks. Participants were administered the first dose of either doxycycline (100 mg for patients weighing ≤50 kg and 200 mg for patients >50 kg) or placebo according to body weight and were always under the supervision of the staff of the FRC during the initial treatment activity.

The participants were then given a single strip pack of the drug containing enough doses for 1 week. To ensure the smooth administration of the daily drug, a care provider (usually a close relative) was identified, and he/she was advised to oversee the daily taking of the medicine and to accompany the patients during all visits to the FRC. Patients were instructed to return to the center at the end of each week (the dates being marked on the patient’s diary by the investigating team) and to return the empty blister pack to confirm the number of tablets likely taken. The patient and the care provider were also asked to record in the diary details of any adverse events (AEs) happening during the period of taking the tablets (42 days), including recording any ADL attacks that occurred. At 3 weeks (day 22) into the treatment, serum transaminase levels were again measured. Urine tests for pregnancy were carried out at baseline and at both 3 and 8 weeks after first taking the drug. A history of any concomitant medications was also repeatedly reported on return visits to the FRC.

### Follow-up.

**Figure 2.**
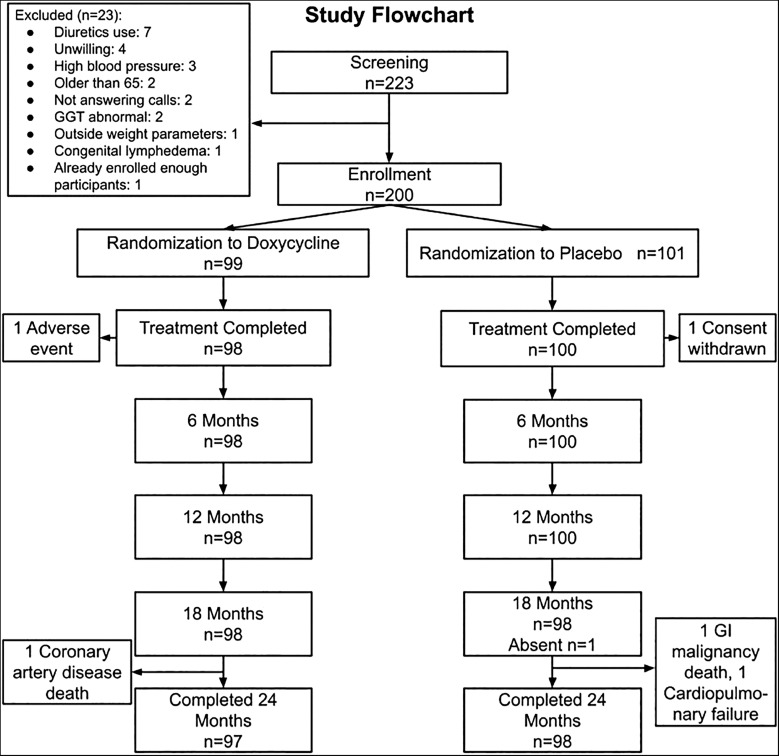
Study flow chart: Enrollment and follow-up of participants. GGT = gamma-glutamyl transferase; GI = gastro intestinal.

Examinations were conducted at 6, 12, 18, and 24 months (Figure 2) with the following procedures carried out: physical examination and vital signs, lymphedema staging of both legs using the Dreyer scale,^16^ and ultrasound skin thickness examination of both legs. Limb circumferences were assessed by taking tape measurements^12^ and volume by LymphaTech three-dimensional photoscans^13^; these studies were repeated, as were clinical photographs, at most of the assessment intervals. Compliance with hygiene measures was assessed repeatedly during the treatment period and at all follow-up points using a defined set of criteria by an independent observer; these included overt cleanliness, interdigital status, and presence of washing materials. In addition to these defined examination time points, subjects were asked to visit the center every 2 months for assessment and recording of the occurrence of ADL and review of each patient’s diary for any unanticipated events. All this information was transferred to electronic case report forms. Adherence to the various components of the EPC were reassessed during these follow-up periods, and retraining was given whenever deemed necessary. The QoL questionnaire was administered again at 12 and 24 months.

## STATISTICAL ANALYSES

Descriptive statistics were calculated for all baseline variables, and bivariate analyses were conducted to determine if randomization yielded treatment and control groups with similar characteristics at baseline. Frequencies were calculated for categorical variables, and Fisher’s exact tests were used. For continuous variables, descriptive statistics were calculated (mean, SD, minimum, maximum, range), and *t*-tests were performed to assess differences between doxycycline and placebo groups. For continuous variables that were non-normal, nonparametric statistics were used. Outcomes were assessed at months 6, 12, 18, and 24 for differences between doxycycline and placebo groups. Differences between doxycycline and placebo groups were compared using two-sided hypotheses with α = 0.05 and 95% CIs. Categorical variables were analyzed using Fisher’s exact tests. Ordinal variables, such as whether lymphedema stage worsened, stayed the same, or improved, were assessed using the Jonckheere–Terpstra test for trend.

Mixed-effects models with a time effect were used to determine whether differences in the measurement endpoints existed between study groups over time. Study group and time were the main effects. Baseline characteristics (e.g., sex, age, and disease history) were included as covariates. Survival analysis techniques were used to assess if differences existed in time until an acute attack between the placebo and treatment groups. Cox proportional hazards regression was used to assess the impact of the treatment adjusted for other covariates.

All analyses were conducted on the intention-to-treat population. Statistical analyses were performed using SAS 9.4 (Cary, NC) and GraphPad Prism (v. 10.0.0, GraphPad Software, San Diego, CA).

### Impact of the COVID pandemic.

This study was carried out in a period when the global COVID-19 pandemic was occurring. Nevertheless, all the WHO-recommended precautions to protect against COVID-19 infection were successfully followed, including maintenance of regular mobile phone contact with patients; these procedures did not interfere in any significant way with following the study protocol.

### Access to data.

The data are accessible at ClinEpiDB: https://clinepidb.org/ce/app/workspace/analyses/DS_f71667fc7f. 253.

## RESULTS

### Trial participants.

As seen in Table 1, sex distribution was biased toward females, with approximately twice as many females in the study as males; this difference, found in both the doxycycline (66 females, 33 males) and placebo (76 females, 25 males) groups likely reflects sociological distinctions in employment, where men frequently labor away from home, outside the district or country. The mean ages of participants in the study groups were similar (52.8 years in the doxycycline group and 52.7 years in the placebo group). The history of participation in MDA programs was not clear for many patients; overall, however, fewer people had been involved in MDA activities than those who had not been involved.

**Table 1:** Baseline data on patients selected to participate in the trial: participant histories

Variable	Doxycycline	Placebo	Total	*P*-Value*
Number of Patients	99	101	200	–
Gender (*N*)				
Male	33	25	58	0.21
Female	66	76	142	
Weight Group (*N*)				
≤50 kg	7	6	13	0.78
>50 kg	92	95	187	
Age				
Mean ± SD	52.77 ± 7.32	52.67 ± 7.62	52.72 ± 7.32	0.93
95% CI	51.31–54.23	51.22–54.13	51.71–53.73	–
Duration of Residence in LF Endemic Area (in years)				
Mean ± SD	51.67 ± 9.48	51.26 ± 10.10	51.46 ± 9.78	0.74
95% CI	49.78–53.56	49.26–53.25	50.10–52.82	–
History of Participation in MDA				
Yes	7	15	22	0.06
No	54	44	98	
Unknown	38	42	80	
Total No. of Patients with ADL Attacks in Past Years				
*N* (% of total group)	50 (52.6%)	33 (34.4%)	83 (43.7%)	0.013
Total No. of ADL Attacks in Past Year	≥50	≥33	≥83	–
Duration (days) of Last ADL Attack				
Range	–	–	–	–
Mean ± SD	6.67 ± 4.74	8.16 ± 5.91	7.42 ± 5.40	0.06
95% CI	5.71–7.64	6.96–9.35	6.65–8.19	–

ADL = adenolymphangitis; LF = lymphatic filariasis; MDA = mass drug administration; *N *= number.

* *P*-value comparing differences between doxycycline and placebo.

### Allocation and completion of the trial.

Of the 200 patients enrolled, 195 completed the 24-month study with its initial 6 weeks of drug or placebo treatment and its long-term follow-up, 97 in the doxycycline group and 98 in the placebo group (Figure 2). There were various reasons for patients not completing the trial, including personal decision to withdraw, physically leaving the area, and death (from causes unrelated to the study). Nevertheless, the number of individuals completing the trial was greater than 95% in each arm.

### Pretrial clinical characteristics.

Having a history of experiencing ADLs during the year prior to the study was significantly more common in the doxycycline group than in the placebo group (Table 1). This difference is particularly notable because ADLs have been associated with worsening of lymphedema and suggests that the group randomized to doxycycline might have been at higher risk for worsening lymphedema than those randomized to placebo. The patients who did have acute attacks during the prestudy year almost always experienced just one or two attacks, and the duration of these attacks was similar (i.e., ∼5–8 days).

Specific clinical measures of lymphedema (i.e., both clinical staging and the assessment of limb size/volume by tape measurements, ultrasound visualization of skin thickness, or LymphaTech scan) were also similar for the two groups prior to initiation of the study (Table 2).

**Table 2:** Baseline data on patients selected to participate in the trial: lymphedema status

Variable	Doxycycline	Placebo	Total	*P*-Value
LE Stage *N*				
Stage 1	0	0	0	–
Stage 2	81	79	160	–
Stage 3	18	22	40	–
LymphaTech Volume (mL)	Median = 2,555	Median = 2,460	Median = 2,482.5	0.63
	Range (1,525–5,155)	Range (1,555–5,145)	Range (1,525–5,155)	
Ultrasound				
Medial Malleolus (cm)	Median = 0.74	Mean = 0.67	Median = 0.73	0.77
	Range (0.19–2.90)	Range (0.12–2.14)	Range (0.12–2.90)	
Lateral Malleolus (cm)	Median = 0.64	Median = 0.50	Median = 0.55	0.08
	Range (0.14–1.63)	Range (0.12–1.73)	Range (0.12–1.73)	
Circumference (cm), Mean (Range)				
10 cm	25.3 (20.0–35.0)	25.1 (20.0–32.0)	25.5 (20.0–35.0)	0.51
12 cm	25.9 (18.0–40.0)	25.7 (19.0–44.0)	25.8 (18.0–44.0)	0.77
20 cm	30.0 (20.0–44.0)	29.4 (21.0–49.0)	29.7 (20.0–49.0)	0.41
30 cm	34.8 (25.0–47.5)	34.8 (27.0–50.0)	34.8 (25.0–50.0)	0.90

LE = lymphedema; *N *= number. Circumferences at specified points of the affected leg (see Materials and Methods section).

### Changes in lymphedema indicators after treatment with doxycycline or placebo.

As seen in Figure 2, almost all of those who entered the trial completed the full course of the study. In addition, there was excellent compliance with the prescribed hygiene protocol during the 2-year study, as inferred directly from review of a “washed and clean limb” indicator assessed at each time point. Essentially 100% of all patients—both those treated with 6 weeks of doxycycline and those receiving only placebo before their almost 2-year follow-up—had washed and clean limbs at all evaluation points, regardless of the treatment group they were in (data not shown). Similarly, changes in the specific parameters associated with the measures of lymphedema evaluated during the study period were essentially equivalent in both groups (Table 3). These measures included leg circumference assessed at four points, skin thickness measured by ultrasound at two points, and limb volume recorded by photo digital scanning (LymphaTech). However, although the two groups were similar in the number of ADL events experienced during each of the years of the 24-month study period (Table 4), there were not only progressive reductions in the number of ADL events (compared with prestudy historical figures) for both groups but also significant decreases from baseline in the *duration* (i.e., shortening) of the ADL episodes in both groups. Interestingly, for the doxycycline group the significant reductions occurred during year 1 of the study (with no further decrease in year 2); for the placebo group, the significant decrease in the duration of ADL episodes was seen not during the first year but during the second year, at which time ADL durations were comparably reduced in both treatment groups. In addition, the affected limbs were subjectively described by both groups of patients as feeling “lighter” after the beginning of the trial and as giving them an increase in their general mobility, with a consequent increase in the ability to carry out their regular daily activities.

**Table 3:** Post-treatment data: Lymphedema measurements of the study participants

Lymphedema measurements	Baseline	6 Months (% change)	12 Months (% change)	24 Months (% change)	*P*-Value (doxycycline vs. placebo–24 months)
Doxycycline					
Circumference (cm)	Mean ± SD				*t*-Test
@10 cm	25.3 ± 2.4	−0.3% ± 0.04	−0.4% ± 0.04	−1.5% ± 0.04	0.09
@12 cm	25.9 ± 4.2	−0.9% ± 0.05	−0.7% ± 0.05	−0.1% ± 0.06	0.04
@20 cm	30.0 ± 5.1	−1.6% ± 0.06	−1.2% ± 0.06	−0.9% ± 0.05	0.63
@30 cm	34.8 ± 4.3	−0.5% ± 0.04	−0.7% ± 0.04	−0.3% ± 0.04	0.70
Skin Thickness (cm) (ultrasound)	Mean ± SD				
@Medial Malleolus	0.8 ± 0.4	0.01% ± 0.3	12.1% ± 0.3	19.0% ± 0.4	0.67
@Lateral Malleolus	0.7 ± 0.3	0.3% ± 0.3	9.0% ± 0.4	14.8% ± 0.4	0.62
Volume (mL)	Mean ± SD				
LymphaTech	2,700 ± 694	1.7% ± 0.1	0.8% ± 0.1	−2.0% ± 0.1	0.49
Placebo					
Circumference (cm)	Mean ± SD				
@10 cm	25.0 ± 2.5	−0.8% ± 0.04	−1.3% ± 0.05	−2.0% ± 0.04	
@12 cm	25.7 ± 4.7	−0.8% ± 0.06	−0.1% ± 0.07	−1.0% ± 0.05	
@20 cm	29.4 ± 4.9	−0.7% ± 0.05	−0.3% ± 0.07	−1.0% ± 0.06	
@30 cm	34.8 ± 4.4	−0.1% ± 0.04	0.1% ± 0.06	−0.2% ± 0.04	
Skin Thickness (cm) (ultrasound)	Mean ± SD				
@Medial Malleolus	0.8 ± 0.4	8.2% ± 0.8	18.0% ± 0.7	17.9% ± 0.4	
@Lateral Malleolus	0.6 ± 0.3	7.4% ± 0.4	16.3% ± 0.5	14.5% ± 0.5	
Volume (mL)	Mean ± SD				
LymphaTech	2,653 ± 686	3.3% ± 0.1	3.2% ± 0.1	−1.5% ± 0.1	

**Table 4:** Acute ADL Attacks

Numbers	Doxycycline	Placebo	Total	*P*-Value (doxycycline vs. placebo)
ADL Attacks Reported to Have Occurred During the Year Before the Study				
Total No. of Patients with ADL Attacks in Past Year (% of total group)	50 (52.6%)	33 (34.4%)	83 (43.7%)	0.013
Total No. of ADL Attacks in Past Year	≥50	≥33	≥83	–
ADLs per Person	1–6	1–5	1–6	–
Duration (days) of Last ADL Attack (mean ± SD)	6.67 ± 4.74	8.16 ± 5.91	7.42 ± 5.40	0.06
95% CI	5.71–7.64	6.96–9.35	6.65–8.19	–
ADL Contemporaneously Recorded During First Year of the Study				
Total No. of Patients with ADLs in Months 1–12 (% of total group)	15 (15.1%)	15 (14.7%)	30 (14.9%)	–
Total No. of ADL Attacks in Months 1–12 (% of total group)	17 (17.3%)	16 (16%)	33 (16.7%)	–
ADLs per Person	1–3	1–2	1–3	–
Duration (days) of ADL Attacks in Months 1–12 (mean ± SD)	4.93 ± 2.12	6.46 ± 4.01	5.64 ± 3.18	0.03
95% CI	–	–	–	–
ADL Contemporaneously Recorded During Second year of the Study				
Total No. of Patients with ADLs in Months 13–24 (% of total group)	10 (10.1%)	13 (12.8%)	23 (11.4%)	0.66
Total No. of ADL Attacks in Months 13–24 (% of total group)	13 (13.4%)	16 (16.3%)	29 (14.9%)	0.81
ADLs per Person	1–3	1–3	1–3	–
Duration (days) of ADL Attacks in Months 13–24 (mean ± SD)	4.30 ± 3.06	4.08 ± 1.24	4.18 ± 2.20	–
95% CI	–	–	–	–

ADL = adenolymphangitis.

### Changes in lymphedema stage.

**Figure 3.**
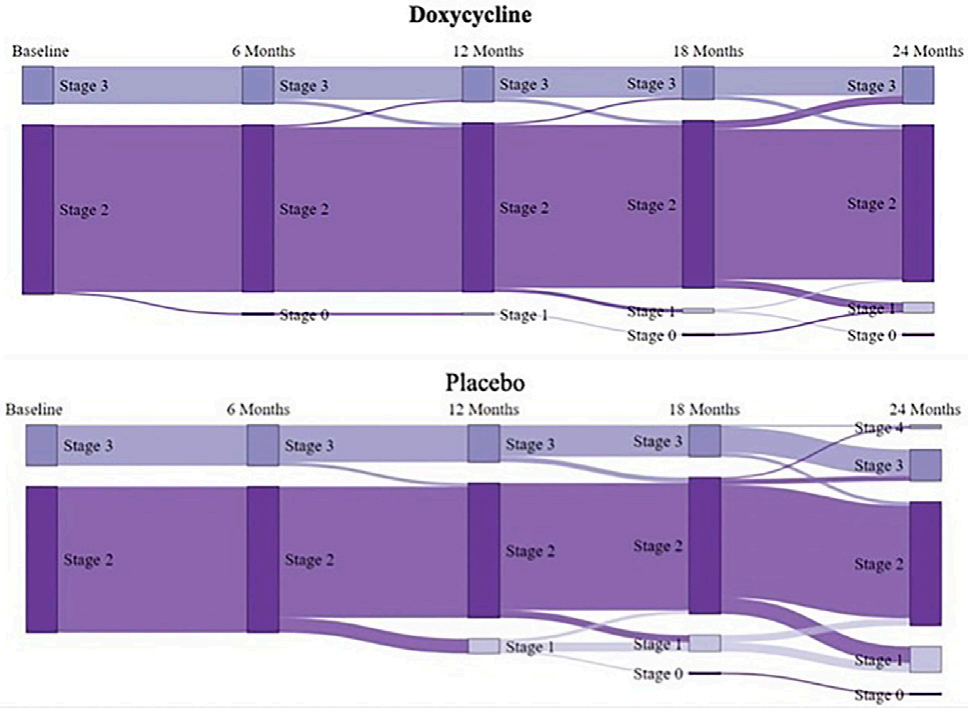
Sankey plots: Improvement of lymphedema stages during follow-up.

**Figure 4.**
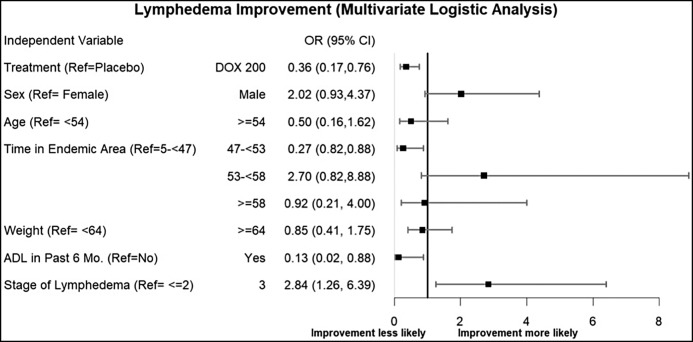
Lymphedema improvement: Multivariate logistic analysis. ADL = adenolymphangitis; DOX = doxycycline; Mo. = month(s); OR = odds ratio; Ref = reference.

The primary endpoint for success in the present study was defined as the demonstration of a benefit from adding a 6-week course of doxycycline to standard EPC management of filarial lymphedema, specifically in improving the stage of patients’ lymphedema. At every time point of the study, each patient’s lymphedema stage was assessed, compared with their pretreatment lymphedema stage, and then determined to have either improved (lower lymphedema stage), worsened (higher lymphedema stage), or remained unchanged. Table 5 shows that over the 24 months of the study 10.3% of the patients receiving doxycycline improved, 85.6% showed no change, and 4% worsened. For those in the placebo group, the numbers were similar (20.6% improved, 76% had no change, and only 3% worsened). Although modest lymphedema improvement over time was evident in both groups of study patients, most remarkable were both the lack of disease progression and the broad homogeneity of lymphedema responses among the patients, as can be seen clearly in the Sankey plot of Figure 3. This Sankey plot shows the flow of participants into and out of the various stages via the arrows, which have a width proportional to the number of persons (i.e., the larger the width, the greater the number of participants who moved to or from a given stage). Regression analysis (Figure 4) identified associations between having stage 3 lymphedema and being more likely to show lymphedema *improvement* during the study period; on the other hand, a statistically significant association was found where having lived in an endemic area between 47 and 53 years was associated with a lower likelihood of lymphedema stage improvement relative to having lived in an endemic area between 5 and 47 years.

**Table 5:** Changes in patients’ lymphedema stage during study

Treatment arm	Month 6	Month 12	Month 24
	*n* (%)	*n* (%)	*n* (%)
Doxycycline			
Improvement*	1 (1.02)	3 (3.06)	10 (10.31)
No Change^†^	97 (98.98)	94 (95.92)	83 (85.57)
Worsening^‡^	0 (0)	1 (1.02)	4 (4.12)
Placebo			
Improvement*	0 (0)	10 (10)	20 (20.62)
No Change^†^	100 (100)	90 (90)	74 (76.29)
Worsening^‡^	0 (0)	0 (0)	3 (3.09)
*P*-Value	0.4949	0.0514	0.0582

Clinical evaluations at visits at 6 months, 12 months, and 24 months.

* Decrease in lymphedema stage.

^†^ No change in lymphedema stage.

^‡^ Increase in lymphedema stage.

Further, having an ADL episode in the past 6 months was associated with a lower likelihood of improvement in lymphedema stage compared with not having an ADL in the past 6 months. Also, a statistically significant association was observed between being in the doxycycline treatment group and being less likely to experience improvement in lymphedema stage.

### Quality of life.

**Figure 5.**
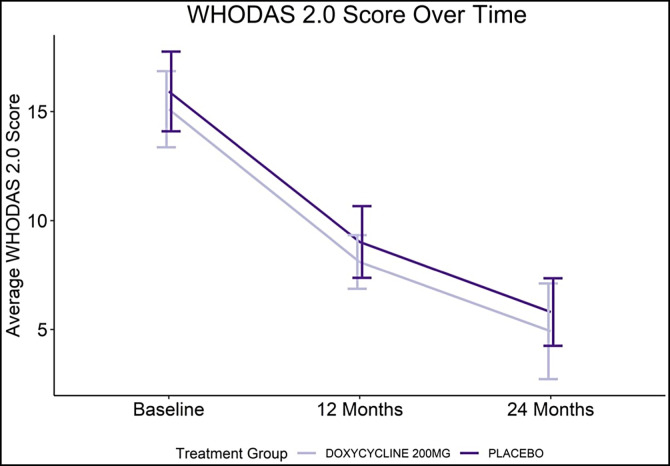
WHODAS 2.0 score metrics. Error bars shown depict the 95% confidence interval associated with the WHODAS point estimate.

**Figure 6.**
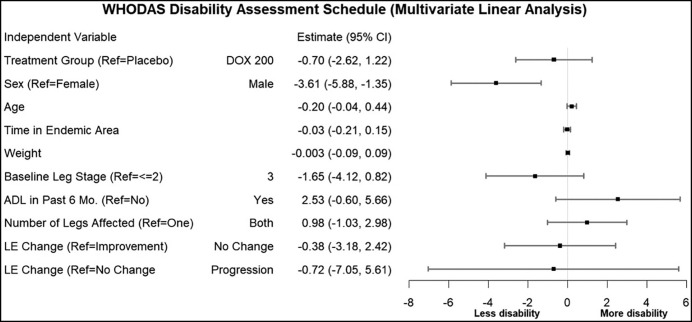
WHODAS disability assessment schedule: Regression analysis. ADL = adenolymphangitis; DOX = doxycycline; LE = lymphoedema; Mo. = month(s); Ref = reference. Error bars shown depict the 95% confidence interval of the odds ratio for the corresponding independent variable.

Over the 24-month study period, significant improvement was documented in the patients’ quality of life, as determined by WHODAS 2.0 metrics (Figure 5). A progressive fall in the disability score (reflecting improved QoL) was seen equally in both doxycycline- and placebo-treated patients. As indicated in the regression analysis (Figure 6), the most important factor associated with this improved QoL (i.e., less disability) was being male, whereas there were trends toward decreased QoL (more disability) both in those with ADL in the 6 months prior to each assessment point and in those who had both legs affected by lymphedema.

### Adverse events.

During the drug-administration phase of the trial (initial 6 weeks) when doxycycline or placebo was given to the patients, AEs were actively monitored by health personnel. The AEs at that time were generally mild and equivalent for the two groups, except for vomiting, which was seen only in the doxycycline group (Table 6). Thereafter, identification of AEs was recorded both when physicians were notified and when all scheduled visits took place. Other than serious AEs that were unrelated to the study treatment, the only AEs requiring hospitalization were attacks of ADL, and these hospitalizations occurred only in the placebo group; no patient in the doxycycline group required such hospitalization.

**Table 6:** Most frequently reported adverse events/serious adverse events during the study

Treatment Group	Doxycycline	Placebo	Total
	*n* (%)	*n* (%)	*N* (%)
Elevated Liver Enzyme Levels	6 (55)	5 (45)	11 (100)
Hospitalization Due to ADL	0 (0)	8 (100)	8 (100)
Upper Respiratory Infection	3 (37.5)	5 (62.5)	8 (100)
Vomiting/Emesis	7 (100)	0 (0)	7 (100)
Head Pain/Headache	2 (33)	4 (67)	6 (100)
Stomach Pain/Stomachache	1 (25)	3 (75)	4 (100)
Fever	1 (33)	2 (67)	3 (100)
Heartburn	1 (50)	1 (50)	2 (100)
Coronary Artery Disease	3 (75)	1 (25)	4 (100)
COVID-19	0 (0)	3 (100)	3 (100)
Blood Pressure Increased	1 (50)	1 (50)	2 (100)
Known Hypertensive	1 (50)	1 (50)	2 (100)
Hospitalization for Cataract Surgery	0 (0)	2 (100)	2 (100)

ADL = adenolymphangitis.

## DISCUSSION

The WHO’s GPELF includes a requirement for national programs to ensure that lymphedema care be available for affected individuals.^3^^,^^4^ Although the EPC does afford many such individuals both relief and some element of improvement,^2^^,^^5^ it is still necessary to continue to seek additional, more effective treatments if possible. Therefore, after earlier promising reports of clinical studies in Ghana,^6^ where 6 weeks of doxycycline was seen to improve cases of lymphedema even in the absence of active filarial infection,^7^ it was incumbent on the filariasis community to explore this regimen as a possible annual or biennial treatment adjunct to improve chronic lymphedema management. Subsequent research findings in both experimental and human filarial infections^17^^,^^18^ suggested that this tetracycline-class drug might have a positive effect by inhibiting the lymphatic endothelium–derived angiopoetic factors thought to underlie the pathogenesis of lymphedema.^19^^,^^20^

Results in the present study, however, generally failed to support these earlier studies’ outcomes and their practical implications for the usefulness of doxycycline as a field-based treatment to be recommended for lymphedema patients—at least for those with lymphedema of stages 1–3. The reasons for this difference in findings from the earlier Ghana studies are not easily discerned but could include any of the following: 1) our current study patients coming from an endemic area of India that has received many more rounds of MDA treatment of LF than the patients in the earlier studies in Ghana, so that these patients lived in very different epidemiological situations with much less exposure to potential reinfection; 2) other environmental differences that exist between the two sets of studies (e.g., the population in the present study being semi-urban, whereas the Ghanaian populations lived in more forest-rural environments); 3) additional local factors at play in the two populations, such as the types of patients’ secondary infections, the timing of peak parasite transmission, the lifestyles of the patients, and even differences in the species of offending parasites involved (*W. bancrofti* versus *B. malayi*); and 4) potentially distinct differences in the effectiveness of the programmatic implementation of the EPC measures between the Ghanaian and Indian study populations.

The parameters monitored in the present study were purposefully wide-ranging, including clinical observations (symptoms and signs), physical measurements, qualitative assessment of well-being, adherence to use of the EPC, and compliance with taking the respective drugs. Many of these observations were maintained on a routinely scheduled basis for 2 years, making this study one of the most robust, longitudinal clinical trials yet to be carried out in individual patients with lymphedema of LF. What is particularly important to emphasize, therefore, is that numerous previous observations—and now also the results of this present detailed investigation—have agreed and consistently demonstrated that the recommended EPC is highly beneficial for managing lymphedema in patients with early LF disease (stages 1–3).^21^^,^^22^

Furthermore, because variations in the presentations of lymphedema are usually more evident in those with higher stages of lymphedema, there is still the important question of whether doxycycline might possibly be useful for managing LF lymphedema of stages greater than 3. The present study was not powered to include such patients, but it should still be emphasized that implementation of *EPC alone* has already been shown to be both relevant and of value in the treatment regimens for patients with these higher stages of lymphedema.^21^ Adenolymphangitis events are recognized as particularly important both in the pathogenesis of lymphedema and as a valuable predictor of the progression of, or conversely, the improvement in clinical lymphedema associated with LF.^21^^,^^23^ Although data on ADLs in the present study population during the year *prior* to the LEDoxy trial were available only through historical recall, the data on ADLs *during* the study were carefully recorded. These well-recorded events provide longitudinal evidence of an association between being *less likely* to see improvement of lymphedema in patients who had at least one ADL recorded during the 6 months prior to the follow-up assessment—in contrast to those with no ADL during that prior 6-month period (Figure 4). This association between recurrent ADLs and less lymphedema improvement might also explain an otherwise puzzling study result in Table 5, where at 12 and 24 months, there were marginally significant (*P* = 0.0514 and *P* = 0.0582, respectively) differences between the placebo and treatment groups in limb stage change, but counterintuitively, the placebo participants experienced more improvement than the treatment group. We suggest that the explanation for this difference is not any negative effect of doxycycline treatment, but rather the result of the random assignment of participants with more extensive histories of ADL to the doxycycline treatment group rather than the placebo group (as seen in Tables 1, 2, and 4).

Otherwise, similar numbers of patients in both the doxycycline and placebo groups had ADLs during the 2 years of the study, and for each group, approximately equal numbers occurred during the first year and second year. These per-year numbers were appreciably lower than the numbers of subjects reporting ADL from the year prior to the study. Similarly, in addition to these “soft data” showing a decreased incidence of ADL during the study, the duration of the ADL episodes was seen to decrease significantly over the 2 study years, most rapidly in the doxycycline group, but equally in the placebo group by the second year of the study. These findings were also reflected clinically in the subjective descriptions of limb lightness and lesser disability in all patients after their 2-year EPC management program, regardless of whether they received doxycycline or placebo treatment.

The findings in the present study (and the related studies carried out in parallel)^9^ provide strong, practical endorsements of the use of the EPC for lymphedema self-care in endemic and post-endemic LF communities. Although such an approach has already been advocated by previous investigators, these present studies arguably constitute the largest and most-detailed endorsement compared with other studies carried out to date that demonstrate the value of EPC. What is of most practical importance now is that national health systems ensure that the support needed to create and sustain the necessary mechanisms is in place for these patients so they can continue their self-care activities. It is common in many countries for these lymphedema patients to let their daily self-care procedures lapse unless there is an active support system in place. Understanding the reasons why patients fail to maintain their care and what their personal opinions are about the usefulness of the EPC in their lives are essential for developing and maintaining more efficient and functional care systems for those affected by this debilitating condition.

## CONCLUSION

This detailed study involving 200 patients with lymphedema in an essentially brugian filariasis–endemic area of southern India indicates that administering 200 mg doxycycline daily for 6 weeks provided little, if any, additional clinical benefit above that already provided through careful adherence to the recommended WHO EPC. Importantly, the study also demonstrated the significant value that the EPC had on both the QoL and the clinical course of lymphedema for trial participants, regardless of whether they received doxycycline or placebo at the beginning of the 24-month study period. As the study was one of a suite of investigations in different country settings using a similar protocol to assess the potential benefits of doxycycline as an adjunct to the EPC and because other study sites included communities in rural West Africa, coastal East Africa, and South Asia, it is likely that any differences in study outcomes that result from specific environmental or social confounders will become clearer when the results of all these individual studies are compared.

## References

[1] Mackenzie CD Mante S, 2021. Caring for patients in the global programme to eliminate lymphatic filariasis. Int Health 13 *(* Suppl 1 *):* S48–S54. 10.1093/inthealth/ihaa080 PMC7753172 33349884

[2] Mackenzie CD Mandara WL Mwakitalu E Dogan N Roundworms: A Survey from Past to Present. London: IntechOpen, 158–160.

[3] World Health Organization , 2023. *Lymphatic Filariasis*. Available at: https://www.who.int/news-room/fact-sheets/detail/lymphatic-filariasis. Accessed September 10, 2024.

[4] World Health Organization , 2021. *Lymphatic filariasis: Managing Morbidity and Preventing Disability: An Aide-Mémoire for National Programme Managers*. 2nd ed. Available at: https://www.who.int/publications/i/item/lymphatic-filariasis-managing-morbidity-and-preventingdisability-an-aide-mémoire-for-national-programme-managers-2nd-ed. Accessed September 10, 2024.

[5] Stocks ME Freeman MC Addiss DG, 2015. The effect of hygiene-based lymphedema management in lymphatic filariasis-endemic areas: A systematic review and meta-analysis. PLoS Negl Trop Dis 9: e0004171. 26496129 10.1371/journal.pntd.0004171 PMC4619803

[6] Debrah AY , 2006. Doxycycline reduces plasma VEGF-C/sVEGFR-3 and improves pathology in lymphatic filariasis *.* PLoS Pathog 2: e92. 17044733 10.1371/journal.ppat.0020092 PMC1564427

[7] Mand S , 2012. Doxycycline improves filarial lymphedema independent of active filarial infection: A randomized controlled trial. Clin Infect Dis 55: 621–630. 22610930 10.1093/cid/cis486 PMC3412691

[8] Wan-Sulaiman WA Kamtchum-Tatuene J Mohamed MH Ramachandran V Ching SM Sazlly-Lim SM Hashim HZ Inche-Mat LN Hoo FK Basri H, 2019. Anti-*Wolbachia* therapy for onchocerciasis & lymphatic filariasis: Current perspectives. Indian J Med Res 149: 706–714. 31496523 10.4103/ijmr.IJMR_454_17 PMC6755775

[9] Horton J , 2020. The design and development of a multicentric protocol to investigate the impact of adjunctive doxycycline on the management of peripheral lymphoedema caused by lymphatic filariasis and podoconiosis. Parasit Vectors 13: 155. 32228663 10.1186/s13071-020-04024-2 PMC7106687

[10] Krishnasastry ST Mackenzie CD Sadanandan R, 2022. Scaling-up filariasis lymphoedema management into the primary health care system in Kerala State, southern India: A case study in healthcare equity. Infect Dis Poverty 11: 9. 35042539 10.1186/s40249-022-00936-6 PMC8764796

[11] Agrawal VK Sashindran VK, 2006. Lymphatic filariasis in India: Problems, challenges and new initiatives. Med J Armed Forces India 62: 359–362. 27688542 10.1016/S0377-1237(06)80109-7 PMC5034168

[12] Scheer R Crofton E Andrews N, 2021. The effect of limb position on the reliability of leg circumference measurements in patients diagnosed with lower limb lymphoedema. Support Care Cancer 29: 3183–3189. 33089370 10.1007/s00520-020-05835-w

[13] Yahathugoda C Weiler MJ Rao R Silva LD Dixon JB Weerasooriya MV Weil GJ Budge PJ, 2017. Use of a novel portable three-dimensional imaging system to measure limb volume and circumference in patients with filarial lymphedema. Am J Trop Med Hyg 97: 1836. 29141750 10.4269/ajtmh.17-0504 PMC5805069

[14] World Health Organization , 2010. *Measuring Health and Disability: Manual for WHO Disability Assessment Schedule (WHODAS 2.0).* Üstün TB, Kostanjsek N, Chatterji S, Rehm J, eds. Available at: https://www.who.int/standards/classifications/international-classification-of-functioning-disability-and-health/who-disability-assessment-schedule. Accessed September 10, 2024.

[15] Axelsson E Lindsäter E Ljótsson B Andersson E Hedman-Lagerlöf E, 2017. The 12-item self-report World Health Organization Disability Assessment Schedule (WHODAS) 2.0 administered via the internet to individuals with anxiety and stress disorders: A psychometric investigation based on data from two clinical trials. JMIR Ment Health 4: e58. 29222080 10.2196/mental.7497 PMC5741825

[16] Dreyer G Addiss D Dreyer P Noroes J, 2002. Basic Lymphoedema Management, Treatment and Prevention of Problems Associated with Lymphatic Filariasis. Hollis, NH: Hollis Publishing Company.

[17] Taylor MJ Bandi C Hoerauf A, 2005. *Wolbachia* bacterial endosymbionts of filarial nematodes. Adv Parasitol 60: 245–284. 16230105 10.1016/S0065-308X(05)60004-8

[18] Turner JD Mand S Debrah AY Muehlfeld J Pfarr K McGarry HF Adjei O Taylor MJ Hoerauf A, 2006. A randomized, double-blind clinical trial of a 3-week course of doxycycline plus albendazole and ivermectin for the treatment of *Wuchereria bancrofti* infection. Clin Infect Dis 42: 1081–1089. 16575724 10.1086/501351

[19] Coulibaly YI , 2009. A randomized trial of doxycycline for *Mansonella perstans* infection. N Engl J Med 361: 1448–1458. 19812401 10.1056/NEJMoa0900863 PMC3410935

[20] Weinkopff T Mackenzie C Eversole R Lammie PJ, 2014. Filarial excretory-secretory products induce human monocytes to produce lymphangiogenic mediators. PLoS Negl Trop Dis 8: e2893. 25010672 10.1371/journal.pntd.0002893 PMC4091784

[21] Shenoy RK Kumaraswami V Suma TK Rajan K Radhakuttyamma G, 1999. A double blind placebo controlled study of the efficacy of oral penicillin, diethylcarbamazine or local treatment of the affected limb in preventing acute adenolymphangitis in lymphoedema caused by brugian filariasis. Ann Trop Med Parasitol 93: 367–377. 10656038 10.1080/00034989958366

[22] Shenoy RK Suma TK Rajan K Kumaraswami V, 1998. Prevention of acute adenolymphangitis in brugian filariasis: Comparison of the efficacy of ivermectin and diethylcarbamazine, each combined with local treatment of the affected limb. Ann Trop Med Parasitol 92: 587–594. 9797832 10.1080/00034989859285

[23] Mackenzie CD Kaminsky R Geary TG Selzer PM Human and Animal Filariasis: Landscape, Challenges, and Control. Hoboken, NJ: John Wiley & Sons, 10–52.

